# Botanical Therapeutics: Phytochemical Screening and Biological Assessment of Chamomile, Parsley and Celery Extracts against A375 Human Melanoma and Dendritic Cells

**DOI:** 10.3390/ijms19113624

**Published:** 2018-11-16

**Authors:** Corina Danciu, Istvan Zupko, Andrea Bor, Anja Schwiebs, Heinfried Radeke, Monica Hancianu, Oana Cioanca, Ersilia Alexa, Camelia Oprean, Florina Bojin, Codruta Soica, Virgil Paunescu, Cristina Adriana Dehelean

**Affiliations:** 1 Faculty of Pharmacy, “Victor Babeş” University of Medicine and Pharmacy, 300041 Timişoara, Romania; corina.danciu@umft.ro (C.D.); camelia.oprean@umft.ro (C.O.); codrutasoica@umft.ro (C.S.); cadehelean@umft.ro (C.A.D.); 2 Faculty of Pharmacy, University of Szeged, H-6720 Szeged, Hungary; zupko@pharm.u-szeged.hu (I.Z.); andrea.bor@pharm.u-szeged.hu (A.B.); 3 Pharmazentrum frankfurt/ZAFES, Clinic of the Goethe University, D-60590 Frankfurt/Main, Germany; schwiebs@med.uni-frankfurt.de (A.S.); radeke@em.uni-frankfurt.de (H.R.); 4 Faculty of Pharmacy, “Gr.T.Popa” University of Medicine and Pharmacy, 700115 Iasi, Romania; mhancianu@yahoo.com; 5 Banat University of Agricultural Sciences and Veterinary Medicine “King Michael I of Romania”, 300645 Timisoara, Romania; alexa.ersilia@yahoo.ro; 6 Faculty of Medicine, “Victor Babeş” University of Medicine and Pharmacy, 300041 Timişoara, Romania; florinabojin@umft.ro (F.B.); vpaunescu@umft.ro (V.P.)

**Keywords:** chamomile, parsley, celery, antioxidant, anti-inflammatory, A375 human melanoma cells, dendritic cells

## Abstract

Chamomile, parsley, and celery represent major botanical sources of apigenin, a well-known flavone with chemopreventive properties. The aim of this study was to assess the phytochemical composition, antioxidant, and anti-inflammatory potential of methanol extracts obtained from chamomile, parsley, and celery collected from Romania, as well as the biological activity against A375 human melanoma and human dendritic cells. Results have shown that all three extracts are rich in polyphenolic compounds and flavonoids, and they generate a radical scavenger capacity, iron chelation potential, as well as lipoxygenase inhibition capacity. Chamomile and celery extracts present weak antiproliferative and pro-apoptotic properties in the set experimental conditions, while parsley extract draws out significant pro-apoptotic potential against A375 human melanoma cells. Parsley and chamomile extracts affected the fibroblast-like morphology of the screened tumor cell line. On the other hand, chamomile and celery extracts abrogated the expansion of LPS-activated dendritic cells, while the metabolic activity was attenuated by stimulation with celery extract; chamomile and parsley extracts had no effect upon this parameter. Chamomile and parsley extracts incubation with naive dendritic cells did not trigger cytokine secretion (TNF-alpha, IL-6, IL-10), but celery extract stimulation significantly reduced the anti-inflammatory, cytokine IL-10.

## 1. Introduction

Natural products, in the form of different types of plant extracts or as pure active phytochemicals, have historically been known to offer important therapeutic support for both traditional and well-established medical approaches. It is unanimously accepted that plants and natural products have played a vital role in medicine and health since the beginning of mankind [1,2]. Being aware of the inherent therapeutic potential of phytochemicals, important lines of research have been headed towards this direction, thus, an impressive number of bioactive compounds have been isolated and deeply investigated over the past decades. Nowadays, we can outline that natural products, together with their chemical derivatives, count more than half of the FDA-approved medication and moreover continue to be important sources for the discovery of active drugs [3].

Recent studies have described that the global incidence of cutaneous melanoma continues to grow year after year and, directly correlated to this, the mortality caused by unresectable or metastatic melanoma unfortunately represents a parameter in evolution [4,5]. Moreover, in the field of oncology, but not only, molecules derived from natural sources represent a huge contribution to drug discovery today. It is worth mentioning that a screening of the number of chemotherapeutic agents, in relation to their source, concludes that over 60% of approved drugs are derived from natural molecules. Vincristine, vinblastine, paclitaxel, docetaxel, topotecan, and irinotecan are only some examples of important plant-derived anticancer agents [6,7]. Among the phytochemicals with anticancer properties, flavonoids represent one of the most studied classes of natural compounds [8,9]. A flavonoid that arouses interest to an increased number of research groups, due to its plethora of biological activities, also including anticancer potential, is 4′,5,7-trihydroxyflavone, commonly known under the name of apigenin. Research in the field has assigned apigenin both in vitro and in vivo antiproliferative, pro-apoptotic, and anti-metastatic effects in experimental models of melanoma [10,11,12].

Chamomile, parsley, and celery represent important sources of apigenin, both for daily human intake and for the industrial extraction of this flavone.

Chamomile, with its scientific name *Matricaria chamomilla* L., also known as German chamomile, is an aromatic plant belonging to the Asteraceae family. The Asteraceae Bercht. & J.Presl family is also called Compositae, due to the composite character of flowers within this family. It is one of the largest families comprising more than 23,000 species included in over 1900 genera [13]. It was asserted that the biological activity of different types of extracts is due to the phytochemicals included in the class of flavonoids (apigenin, luteolin, quercetin, patuletin) and essential oils (α-bisabolol and its oxides, azulenes) [14]. The main biological activities include antioxidant, antimicrobial, anti-inflammatory, cytotoxic, antispasmodic, antiviral, and sedative potential [15]. The antiproliferative potential of chamomile extract was described for various cell lines, including human prostate epithelial PZ-HPV-7 cells, human prostate cancer LNCaP, DU145, PC-3 cells, T-47D breast carcinoma, HeLa -cervical adenocarcinoma, HT1080- fibrosarcoma, and RKO-colon carcinoma cells [16].

Parsley and celery are also aromatic plants belonging to the family Apiaceae. Apiaceae Lindl., also known as Umbelliferae Juss. This family represents the 16th-largest family of flowering plants, and comprises approximately 3000–3750 species included in 300–455 genera [17]. Parsley and celery are two important constituents of this family, used both for their culinary and medical benefits. A comprehensive review that presents the ethnopharmacology, phytochemistry, and biological activities of parsley, also known under the scientific name of *Petroselinum crispum* (Mill.) Nym. ex A. W. Hill, concludes that the seed extract has in vitro antioxidant, analgesic, spasmolytic, immunosuppressant, laxative, and diuretic properties [18]. A recent study has shown that extracts obtained from the leaves and stem of English parsley indicate an antioxidant capacity, as well as a protective effect against DNA damage induced by H_2_O_2_. Moreover, the extract has been shown to inhibit the proliferation and migration of MCF7 breast cancer cell line [19]. Celery seeds extracts have been described for their antioxidant, antimicrobial, antiarthritic, and antiulcer potential [20,21]. The group of Mansi et al. have also found that the extract can induce a hypolipidemic effect in rats [22]. Anti-inflammatory, gastro-protective, anti-*Helicobacter pylori* activity, and no toxicologically significant subchronic effects in experimental models using rats, were reported by the group of Powanda et al. [23]. Wild celery oil was assigned with antiproliferative potential against HCT116 human colon carcinoma cells [24].

The aim of this study is to assess the phytochemical composition, and antioxidant and anti-inflammatory potential of some major botanical sources of apigenin—chamomile, parsley, and celery methanolic extracts—as well as their biological activity against A375 human melanoma and human dendritic cells.

## 2. Results

### 2.1. UHPLC Chromatograms of the Investigated Extracts

The main compounds that were identified in all investigated samples belong to the polyphenolic acids and flavone groups. The most important, quantitatively, are included in Table 1.

**Figure 1:**
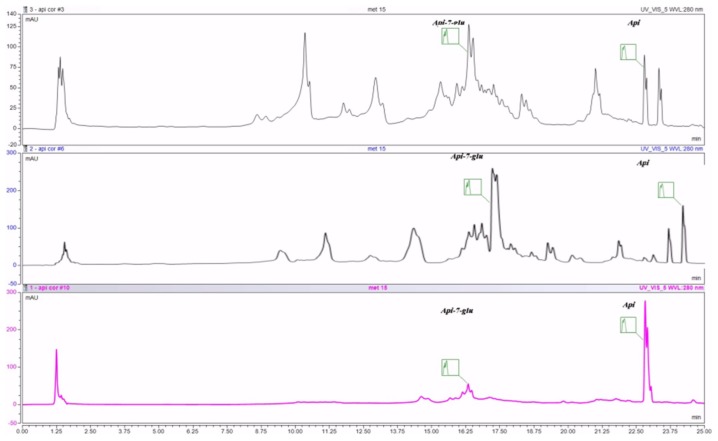
UHPLC chromatogram of the investigated extracts: MC—chamomile flower extract, C- celery seeds extract, P—parsley seeds extract; Api-7-glu—apigenin glucoside, Api—apigenin.

As indicated in the table, each extract has variations with regard to the main compounds, but it is clearly visible that the seeds contain mainly aglycons and less glycosides. UHPLC chromatograms of the investigated extracts are displayed in Figure 1.

### 2.2. Spectrophotometric Quantification of Flavonoids and Total Phenols of the Investigated Extracts

The spectrophotometric quantification of flavonoids and total phenols is presented in Table 2.

### 2.3. Radical Scavenger Capacity of the Investigated Extracts

Radical scavenger capacity was assessed by DPPH and ABTS binding capacity. The color changes noted during testing are included in Table 3. The results are expressed as EC_50_, which represents the efficient concentration to chelate 50% of the free radicals present in the assay environment. These results indicate the extract quantity necessary to be used in order to obtain at least 50% inhibition against free radicals.

### 2.4. Iron Chelation Potential of the Selected Extracts

The bivalent iron is indirectly involved in the occurrence of oxidative stress because it participates in the Fenton reaction by which hydroxyl radicals are generated. The latter exhibit particular chemical reactivity, and may initiate oxidation reactions, in particular, of unsaturated compounds, with damage to the cell membrane structure or other biologically relevant compounds. Therefore, the chelation of ferrous ion greatly decreases the availability of ions for the Fenton reaction, which might explain the antioxidant and protective activity of the investigated extract. Results can be seen in Table 4.

### 2.5. Lipoxygenase Inhibition Activity of Selected Extract

Determination of lipoxygenase inhibition activity was conducted using the modified Maltreud method [25], based on the principle that the polyphenolic compounds present in the extract have the ability to block the action of lipoxygenase that catalyzes oxidation of linoleic acid with a decrease in absorbance at 234 nm. There are two possible mechanisms by which the compounds in the tested extracts can block the enzyme activity, as follows: either by blocking the redox reversible transformation process of Fe^2+^ from Fe^3+^ and, thus, blocking the oxidation of the substrate; or by modifying the spatial structure of the active site or of the enzyme. The lipoxygenase inhibition activity is linear with the concentration. Results can be seen in Table 5. Such results sustain the anti-inflammatory activity of the tested extracts. The EC_50_ value indicates that, for a good inhibitory effect, only 166.32 ± 2.03 mg of MC extract, 86.15 ± 4.82 mg of C extract, and 69.46 ± 1.70 mg of P extract need to be used. There is still a great difference between the EC_50_ calculated for the standard (caffeic acid) and the investigated extracts. This indicates that the standard should be more active than our samples. Nevertheless, the calculated value of EC_50_ proves that all three extracts are active against LOX, and their activity decreases in the following order: P > C > MC. Usually, amounts of ca. 70–170 mg of plant extracts are quite low, taking into account that this is the quantity necessary to inhibit 50% of the lipoxygenase activity.

### 2.6. Antiproliferative Activity of Selected Extracts against A375 Human Melanoma Cell Line

**Figure 2:**
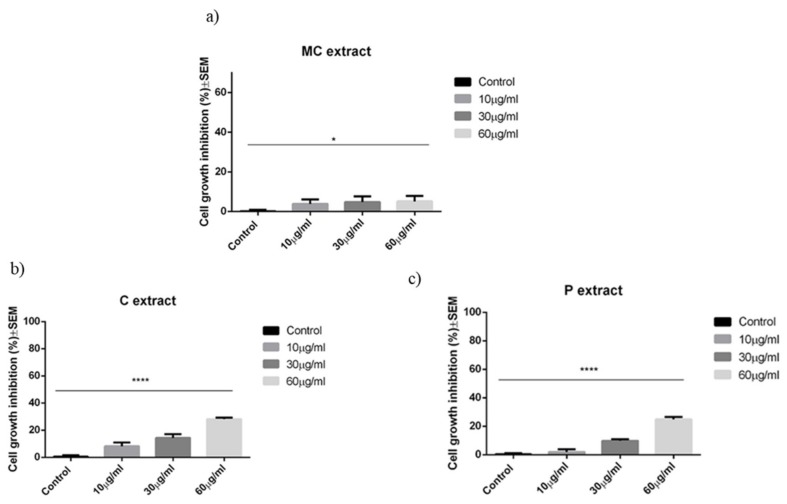
Cell growth inhibition (%) ± SEM against A375 human melanoma cells after 72 h of incubation with (**a**) MC extract; (**b**) C extract; or (**c**) P extract. Statistical significance was assessed by one-way ANOVA with Newman–Keuls post-test for comparison of multiple groups * and **** indicate *p* < 0.05 and *p* < 0.0001 respectively, compared to the control group.

The three extracts show an overall weak antiproliferative activity in the range of tested concentrations, C extract being more active, starting from the concentration of 10 μg/mL. At the highest tested concentration, namely 60 μg/mL, and after a period of 72 h incubation, the C extract led to a cell growth inhibition of 28.1 ± 2.0%, the P extract led to a cell growth inhibition of 24.9 ± 2.9%, while the MC extract led to a cell growth inhibition of 5.17 ± 3.4%. Results can be seen in Figure 2.

### 2.7. Cell Cycle Distribution of A375 Human Melanoma Cells after Incubation with Selected Extracts

**Figure 3:**
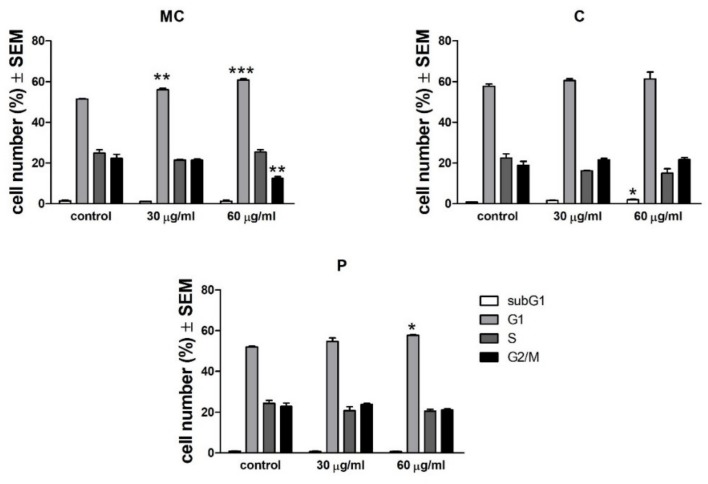
Effect of MC, C, and P extracts on cell cycle phases for A375 melanoma cell line. Statistical significance was assessed by one-way ANOVA with Newman–Keuls post-test for comparison of multiple groups *, ** and *** indicate *p* < 0.05, *p* < 0.01 and *p* < 0.001, respectively, compared to the control group.

**Figure 4:**
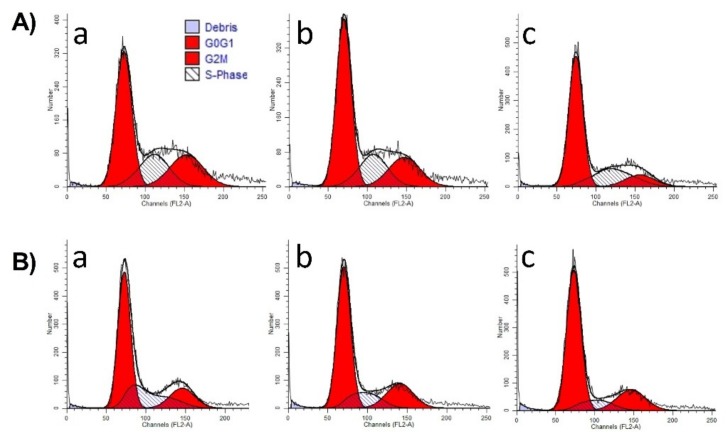

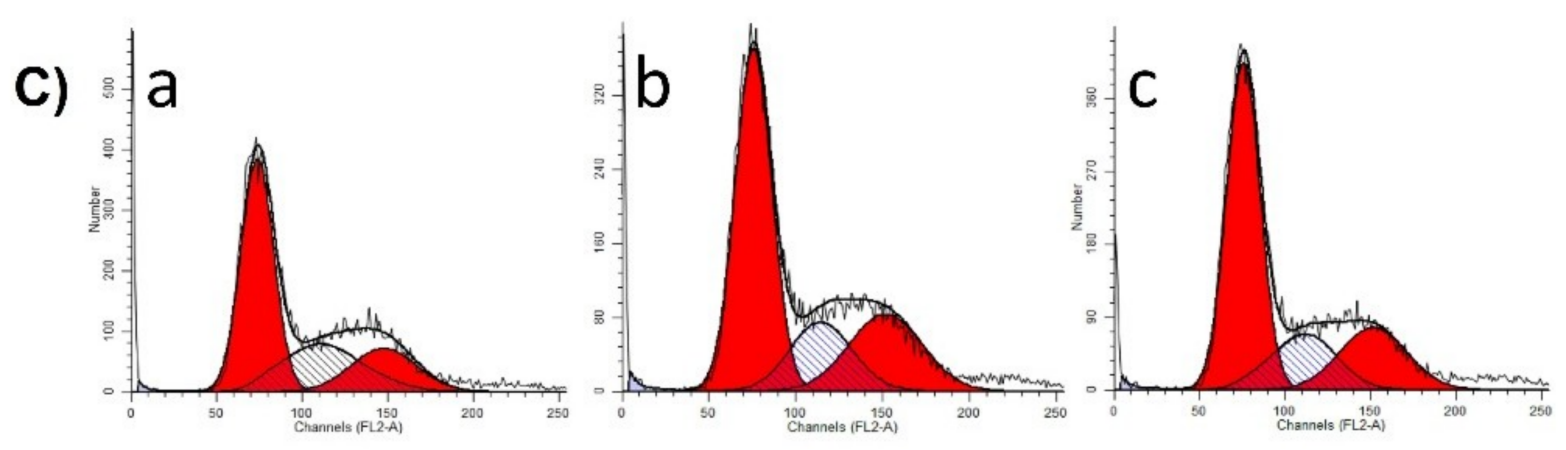
R epresentative histograms that describe the effect of (**A**) MC, (**B**) C, (**C**) P on cell cycle phases of A375 melanoma cell line with the following assignments: (**a**) Control, (**b**) 30 μg/mL, (**c**) 60 μg/mL.

The MC, C, and P extracts at the concentrations of 30 and 60 μg/mL, respectively, did not trigger any significant changes in the distribution of the cells between the phases of the cell cycle. A slightly increased number of cells was detected in G1 phase compared to control for MC extract, while for C and P extracts, a slight subG1 phase accumulation was observed. Results can be seen in Figure 3. Representative histograms of the distribution of the phases of the cell cycle can be seen in Figure 4A–C.

### 2.8. Caspase 3 Activity of A375 Human Melanoma Cells after Incubation with Selected Extracts

**Figure 5:**
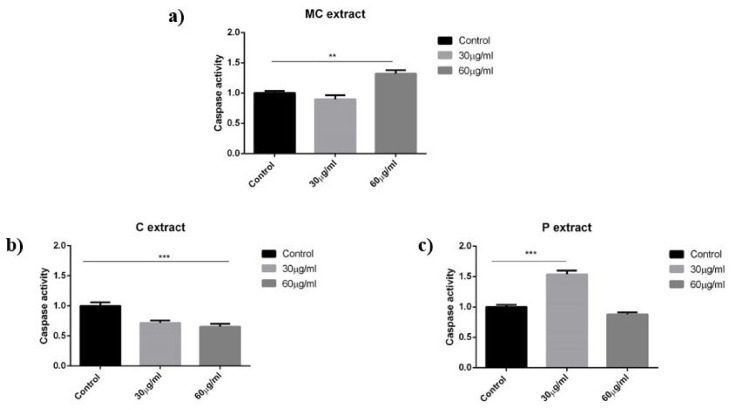
Caspase 3 activity of A375 human melanoma cells after 72 h of incubation with (**a**) MC extract; (**b**) C extract; (**c**) P extract. Statistical significance was assessed by one-way ANOVA with Newman–Keuls post-test for comparison of multiple groups ** and *** indicate *p* < 0.01 and *p* < 0.001, respectively, compared to the control group.

The activity of protein caspase 3 as an effector caspase of apoptosis was analyzed for the three extracts using the same two concentrations, namely 30 and 60 μg/mL. However, at 30 μg/mL, no effect was detected for the MC extract while, at the highest tested concentration of 60 μg/mL, a significant increase of caspase 3 activity was induced as compared with control. Results have shown that, in case of C extract, caspase 3 is not activated in the tested dose range. On the other hand, the P extract in the concentration of 30 μg/mL significantly increased the amount of this protein, which shows the pro-apoptotic potential of P extract at this dose. Interestingly, when 60 μg/mL P extract was used, the amount of caspase 3 decreased, probably as a consequence of necrosis. Results can be seen in Figure 5.

### 2.9. Percentage of Apoptotic Events for A375 Human Melanoma Cell Line after Incubation with Selected Extracts

In order to analyze, more thoroughly, the apoptotic events, annexin V-PI staining was used, in order to have data about early apoptosis, late apoptosis, and necrotic events. Correlated to the abovementioned findings, the study showed that P extract at the concentration of 60 μg/mL can induce phenomena of early and late apoptosis, as well as necrosis, compared to control cells. Results can be seen in Table 6.

### 2.10. Caspase 2 and p53 Expression for A375 Human Melanoma Cell Line after Incubation with Selected Extracts

**Figure 6:**
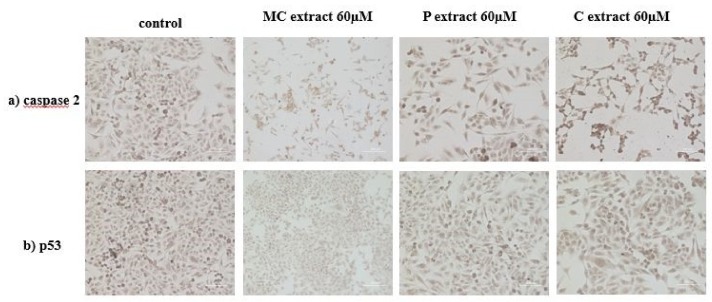
Immunocytochemical evaluation of A375 human melanoma cells after incubation with selected extracts at a concentration of 60 μM; (**a**) caspase 2 evaluation; (**b**) p53 evaluation (Magnification 10×; Scale 200 μm).

Immunocytochemistry was performed in order to detect the expression of caspase 2 as a possible inductor of caspase 3. This technique also allowed for observation of the number of cells along with their morphology. Regarding the marker expression, a similar relative ratio, in control and cells incubated with 60 μg/mL MC and C extract, could be detected. Increased caspase 2 expression, in the case of cells treated with 60 μg/mL P extract, could be observed. A reduced number of cells compared to control, in the case of cells treated with selected extracts, was noticed. The morphology of A375 human melanoma cells is fibroblast-like. Incubation with 60 μg/mL C extract did not generate changes in the morphology of cells. A cytoplasm reduction and elongated cells were present upon incubation with 60 µg/mL MC extract. Incubation with 60 μg/mL P extract led to the reduction of the volume of the cytoplasm, as well as elongated and thinned cell shape. The expression of cellular tumor antigen p53 in A375 human melanoma cells was also checked after incubation with the three extracts in the concentration of 60 μg/mL. A similar expression of p53 marker could be noticed in all samples, thus, this tumor suppressor marker is not activated following incubation with the selected extracts. Results can be seen in Figure 6.

### 2.11. Evaluation of the Effect of Selected Extracts on Human Dendritic Cells

**Figure 7:**
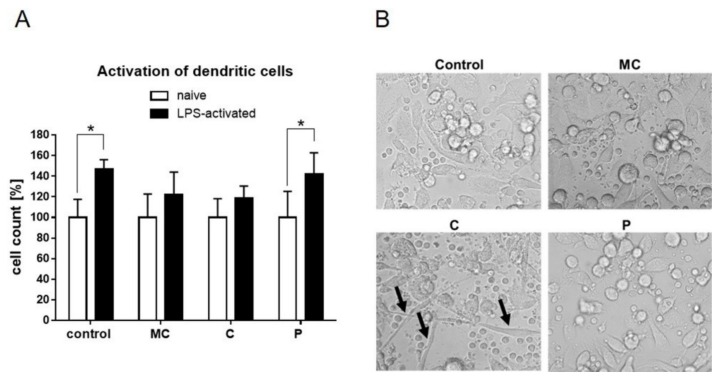
(**A**) Activation of human dendritic cells measured by cell expansion after 24 h stimulation with vehicle, MC extract, C extract, or P extract (60 µg/mL) in the absence (naïve) or presence of LPS. Naïve cells were set to 100%. (**B**) Representative transmitted light microscopic pictures 24 h after extract stimulation (60 µg/mL) of naïve human dendritic cells (Magnification 20×; black arrows indicate endocytic enlargement). (* *p* ≤ 0.05; *n* = 4).

In order to test immunomodulating effects of the extracts, primary PBMCs have been isolated from blood, differentiated into dendritic cells, and stimulated with corresponding amounts of the three extracts. As expected, basic LPS activation of the DCs led to cell activation, indicated by rapid cell expansion within 24 h (Figure 7A). This significant increase was abrogated by parallel stimulation of MC and C extracts, while the P extract had a similar effect compared to control. Transmitted light microscopic pictures of naïve cells treated with the extracts present some enhanced endocytic capacities of C extract-stimulated cells (Figure 7B).

**Figure 8:**
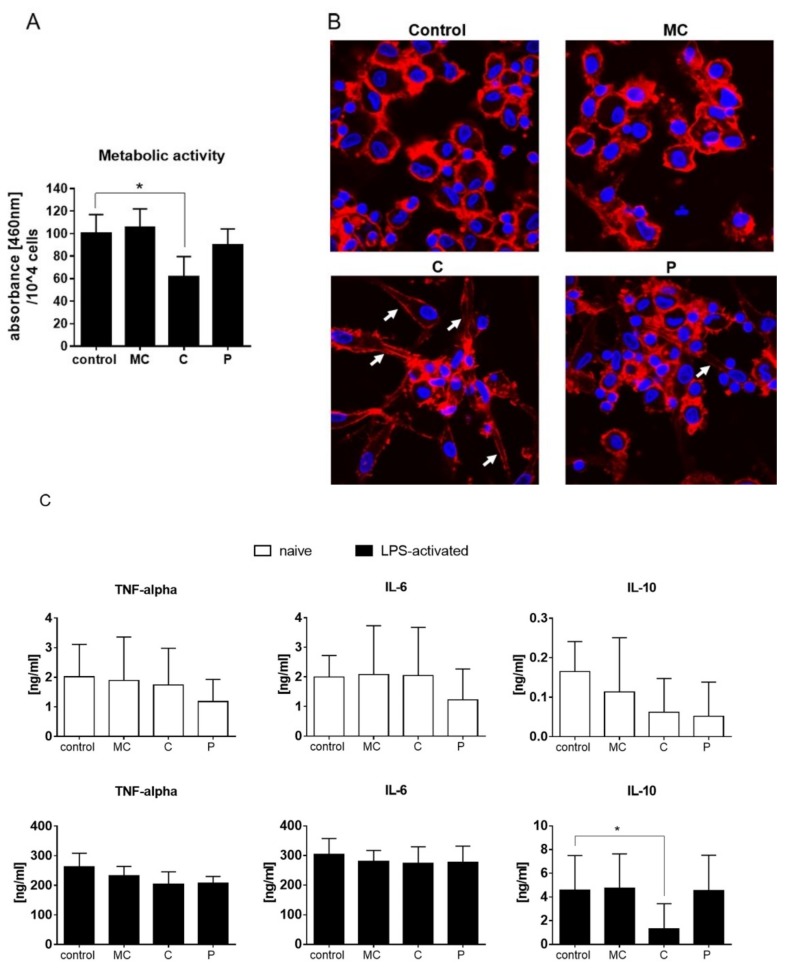
(**A**) XTT assay for metabolic activity of LPS-activated human dendritic cells stimulated with vehicle, MC extract, C extract, or P extract (60 µg/mL) for 48 h. (**B**) Representative confocal microscopic pictures of LPS-activated human dendritic cells treated with vehicle, MC extract, C extract, or P extract (60 µg/mL) for 48 h (blue = DAPI (Nulcei), red = phalloidin (F-actin); Magnification 63X; White arrows indicates endocytic enlargement). (**C**) Quantification of cytokines in the supernatant of LPS-activated human dendritic cells stimulated with vehicle, MC extract, C extract, or P extract (60 µg/mL) for 24 h (* *p* ≤ 0.05, *n* = 4).

XTT assays were performed in order to elucidate if reduced LPS activation was due to reduced metabolic activity of the cells. Metabolic activity of active human DCs was attenuated by stimulation with C extract, while MC and P extract had no effect on metabolic activity (Figure 8A). Confocal microscopy at 48 h after stimulation revealed typical clustering of cells upon LPS activation (Figure 8B). Clustering was also present in C extract-treated cells although, again, an endocytic enlargement (white arrows) was present, as indicated by membrane staining. Sporadically, those spindle forms were also present in P extract-treated LPS-activated cells. Cytokine secretion was analyzed in order to see if the slightly reduced cell activity of LPS-activated cells had functional consequences. Extract incubation with naïve dendritic cells did not trigger cytokine secretion, indicating that the extracts themselves had no immune reactivity in the given settings. TNF-alpha and IL-6 level induction in DC supernatant was similar in all treated groups compared to control (Figure 8C). The level of the anti-inflammatory cytokine IL-10 was significantly reduced upon C extract stimulation.

## 3. Discussion

All three investigated vegetal products are postulated in the literature as major botanical sources of apigenin but, according to their scientific classification, chamomile belongs to the Magnoliophyta clade, Asteraceae family, while parsley and celery belong to the Angiosperms clade, Apiaceae family. This is the reason why we have decided to discuss the phytochemical composition in these two directions, namely presentation of chamomile extract, and parsley and celery extracts, respectively. The main classes of phytochemicals with biological activity that can be found in the chamomile flowers include volatile oil (which, in turn, is composed by sesquiterpenes azulenogens and non-azulenogens, as well as monoterpenes), flavonoids, coumarins, terpenoids, and mucilages. In a comprehensive review about this medicinal aromatic plant, Srivastava et al. have estimated the presence of approximately 120 secondary metabolites, including 28 terpenoids and 36 flavonoids [26]. From the class of flavonoids, apigenin represents one of the most promising compounds from the point of view of bioactivity. Within the vegetal product, the glycosidic form is predominant, while the aglycone can be found in small amounts [27]. It is unanimously accepted that the most usual source of apigenin intake for the human body is represented by chamomile tea [28].

The study analyzed a hydroalcoholic extract based on the idea postulated in the literature that optimum extracts obtained from this vegetal product contain about 50% alcohol [26]. According to the European Pharmacopeia (EP), in order to have a biological effect, the minimum percentage of apigenin 7-glucoside in the flowers should be 0.25% [29]. Our results confirm that the vegetal product complies with the pharmacopoeia provisions. In a similar approach, Fonseca et al. concluded that the percentage of free and glycosylated apigenin in the methanol extract is 106 and 903 μg/g whereas, in the ethanol extract, the amount is 11 and 247 μg/g [30]. Analyzing the amount of pure and conjugated apigenin in different types of extracts, Haghi et al. have observed that the methanol extract leads to the highest amount of pure apigenin [29]. In an aqueous extract, apigenin 7-*O*-glucoside was found to be the major constituent [31].

In a similar approach using different solvents for the extraction of active compounds from the aerial parts in the flowering stage of chamomile (e.g., water, methanol, ethanol), Haghi et al. have concluded that the amount of total phenolic compounds and total flavonoids range in the interval (1.77–50.75 g GAE/100 g in dry material) and (0.82–36.75 g quercetin equivalent (QE)/100 g in dry material), respectively [29].

As revealed in the results section by different assays, it is obvious that the MC extract demonstrates free radical-scavenging potential. The practical approach is that intake of different food supplements or tea based on chamomile flower extract can lead to prevention of an increased number of pathologies related to oxidative stress and, also, prevent cell mutation. In a recent study, Cvetanovic et al. have assessed the antioxidant potential of different types of extracts obtained from chamomile flowers, namely Soxhlet, microwave-assisted, ultrasound-assisted, and subcritical water extraction. They concluded that the best free radical-scavenging ability was generated by subcritical water extraction [32]. The stamp that confirms the antioxidant potential of water and alcohol extracts of this vegetal product was put down also by Al-Ismail et al., who used a linoleic acid and liposome model system, as well as the well-known DPPH free radical-scavenging assay [33]. The phytochemicals that might be involved in this type of effect are the flavonoids, due to their polyphenolic structure; and the terpenoids, due to their double bond system. A recent study showed the protective role of chamomile decoction extract, using a rat model of alcohol-induced injury of gastric mucosa. This potential was assigned to its capacity to reverse the depletion of antioxidant enzymes activity, such as superoxide dismutase (SOD), catalase (CAT), and glutathione peroxidase (GPx) induced by ethanol administration [34]. Moreover, our results confirm that MC extract has a better activity against LOX than against free radicals, suggesting that different mechanisms are involved for such properties. The LOX-inhibiting activity can be correlated with an anti-inflammatory potential, which may decrease the membrane permeability.

In regard to parsley and celery extracts, the majority of research has involved different plant organs, usually leaves, stems, and culture cells [35,36]. Moreover, the essential and fatty oils were mostly investigated. In terms of our choice of vegetal product, the seeds are commonly believed to contain the genuine and simple compounds found in the future plant. The chemical analysis of parsley and celery seed alcoholic extracts revealed that the chosen vegetal material contains flavone aglycons and apigenin glucoside [37]. Other studies indicated the presence of gallic acid, catechins, and its derivatives, in ethanolic leaf extract, which were also present in our samples in small quantities. The authors concluded, then, that polyphenols, and mainly kaempferol derivatives, were responsible for the antioxidant activity [38].

At the same time, the calculated EC_50_ value for all the antioxidant tests allowed us to observe differences in regard to their activity. Taken together, all investigated extracts showed a better scavenger activity against free radicals, a good inhibitory activity against lipoxygenase, but a lower sensitivity against iron. These confirm that the type of bioactive compound is highly important for the activity of the extract. Such claims have been previously discussed by Csepregi et al. [39] in terms of structure–activity relationships, when the presence of certain free hydroxyl groups on the phenolic or flavonoidic skeleton can enhance the antioxidant/scavenger/chelation potential, depending on the assay.

However, the total phenolic quantification is not in direct correlation with the extract antioxidant capacity. There is still a correlation between the flavonoid content, the presence of aglycons, and the antioxidant potential for both parsley and celery samples. Every extract reacts differently depending on the assay, and the ratio and distribution of each component; for example, parsley and celery are more potent as enzyme inhibitors, whereas the chamomile extract acts more strongly as a radical scavenger. Moreover, all extracts show a weaker iron-binding activity. Our results sustain the observations made previously on the antioxidant capacity of parsley and celery extracts [36,40]. Some studies also suggest that the type of solvent used for extraction and the temperature used during processing has a great impact on the chemical composition and the biological implication of the final extract. The current study suggests that the chosen extraction method and solvent ensure a good chemical composition of the investigated extracts.

As discussed in the results section for the selected concentrations, the chamomile extract presents weak antiproliferative and pro-apoptotic potential against A375 human melanoma cell line. In a recent paper, Sak et al. have shown that methanolic extract obtained from German chamomile (*Matricaria recutita* L.) presents a cytotoxic effect against SK-MEL-2 human melanoma cells with an IC_50_ value of 40.7 μg/mL [41]. In a comparative study that analyzed the anticancer activity of chamomile and marigold tea extract on different cancer cell lines, including a human melanoma cell line (Fem-x), the IC_50_ value was >16.67 mg/mL for chamomile extract, while marigold tea extract generated a higher anti-melanoma effect, translated to an IC_50_ value of 0.36 ± 0.12 [42]. On human dendritic cells, chamomile extract had a slight effect on LPS-mediated activation which however, did not change the metabolic activity or cytokine secretion of TNF-alpha, IL-6, or IL-10. 

To the best of our knowledge, until this moment, there are no reports in the literature regarding the effect of celery extract on human melanoma cells. Our study indicates a weak antiproliferative potential against A375 human melanoma cells linear with the concentration. Celery extract was assigned with cytotoxic potential for other cancer cell lines, which gave us the idea to perform a screening for the selected melanoma cell line. For example, in a recent paper, Ahmand et al. have pointed toward the antiproliferative and pro-apoptotic activity of celery seeds extract against BGC-823 human stomach cancer cell line [43]. Methanolic celery extracts have produced cytotoxic events and apoptosis in the case of two different human cell lines, namely, DLA, Dalton’s lymphoma ascites, and L929, mouse lung fibroblast [44]. Polyacetylenes obtained from celery root have shown in vitro cytotoxic effect against five different cancer cell lines [45]. Moreover, methanolic celery extracts have indicated a protective effect against an in vivo model of chemically induced hepatocarcinogenesis [46]. Celery slightly reduced the ability of dendritic cells to proliferate during LPS activation, and led the cells acquire a spindle-like shape. Notably, this effect did not cause reduction in the pro-inflammatory cytokine levels of TNF-alpha and IL-6 but, rather, reduced secretion of the anti-inflammatory IL-10. Thus, celery extract might be a potential source to further enhance active immune responses.

The parsley extract was analyzed by the group of Dorman et al. They have tested the cytotoxic potential of parsley extract obtained from the leaves of *Petroselinum crispum* (Mill.) Nym. ex A. W. Hill against non-cancerous CV1-P fibroblast and A375 human melanoma cells. The MTT assay has shown that cancerous cells were more sensitive to parsley extract treatment, in comparison to normal fibroblasts. The highest tested concentration, namely 2 mg/mL, led to a 50% decrease of the metabolic activity of cancerous cells. The leakage of lactate dehydrogenase, as a sign of disintegration of the cell membrane, increased by 210% for A375 cells and 179% for CV1-P cells. The expression of caspase 3 as a predictor of apoptosis was assessed, but the extract had no effect on the activation of this pro-apoptotic protein [47]. The literature demonstrates that breast cancer cells can also be sensitive to parsley extract. For example, the root extract at a concentration of 500 μg/mL inhibited the DNA synthesis and metabolic activity of MCF712A and MCF7 estrogen receptor-positive benign and malignant mammary cells [48]. Tang et al. have shown that dichloromethane extracts obtained from leaves and stems inhibit proliferation and migration of MCF7 cells [19]. On dendritic cells, parsley extract had no anti- or pro-proliferative effect.

## 4. Materials and Methods

### 4.1. Extracts

Dry chamomile flowers (*Matricaria chamomilla* L.) were purchased from a Romanian cultivator (Iasi, Romania), the material was identified, and a voucher specimen (code Mc10/2016) was deposited at the Department of Pharmacognosy, “Grigore T. Popa” University of Medicine and Pharmacy Iasi, Romania. Flavonoids and polyphenolic acids were isolated from chamomile flowers by hydroalcoholic extraction (methanol 60%), in which the drug extract ratio (RDE) was 2.5 g of plant product per 100 mL of final extract. The fluid extract was concentrated to dry consistency with a Buchi rotary evaporator.

Parsley (*Petroselinum crispum* var. radicosum Miller) and celery (*Apium graveolens* var. radicosum L.) seeds were purchased from specialized providers, and their batch numbers were given to the voucher specimens (code Pc01/2016 for parsley and code Ag05/2016 for celery) which are currently kept along with the chamomile specimen.

After initial testing for certification of its identity, both parsley and celery were extracted with a methanolic solution for which the methanol/water ratio was 60:40. The extracts were obtained on a thermostatic water bath, starting with 2.5 g of powdered plant material. The final solution (100 mL) was leveled in a volumetric flask. After concentration in a rotary evaporator, the extracts were weighted and stored at 4 °C until further testing.

Apigenin ≥99% (HPLC) (CAS Number 520-36-5) was acquired from Sigma-Aldrich, Steinheim, Germany.

### 4.2. Sample Coding 

For easier reference in the present paper, all extracts were coded as follows: MC for chamomile extract, P for parsley extract, and C for celery extract. The plant name has been checked against http://www.theplantlist.org.

### 4.3. RP-UHPLC Quantification

UPLC was performed with a Thermo UltiMate3000 gradient chromatograph equipped with a quaternary pump controlled by Chromeleon interface, an autosampler, and multidiode array detector (DAD). Solvents were filtered using a Millipore system, and analysis was performed on an Accucore XL C18 column (150 × 4, 6 × 4). The mobile phase was acetonitrile (A) and water containing 0.1% acetic acid (B), and the gradient was 10–23% (A) in 5 min; 23% (A) isocratic for 10 min and then 23–35% (A) in 12 min; 35–70% (A) for 5 min. The injection volume was 20 µL, and scanning absorbance wavelengths from 240 to 520 nm, typical for phenols. The flow rate increased from 0.2 to 1 mL/min. HPLC grade solvents and double-distilled water were used in the chromatographic studies. All chromatographic experiments were performed at 25 °C.

All polyphenol standards of analytical grade were purchased from Sigma Chemical Co. (Steinheim, Germany) Triplicate injections were used for both samples, and standards and their calibration curves were obtained using the average value.

### 4.4. Spectrophotometric Quantification of Flavonoids

The quantification of total flavonoids from plant material employed its complexation properties with aluminum chloride (AlCl_3_) according to known techniques [49]. An aliquot of the dry extract was solubilized in 1.25 mL water and 0.075 mL 5% sodium nitrite (NaNO_2_). After 6 min, 0.15 mL 10% AlCl_3_ were vortexed together, and then 0.5 mL 1 M sodium hydroxide was added to the mixture. The intensity of the red color was measured at λ = 510 nm.

### 4.5. Total Phenols Quantification

Total phenolic content (TPC) of the extract was estimated according to the method described by Mircea et al. [50]. The sample was prepared by dissolving 6.8 mg in 1 mL methanol and homogenized. Briefly, 400 μL of the sample was mixed with 0.2 mL Folin–Ciocalteu reagent, and 3.16 mL distilled water. After 5 min incubation, 0.6 mL 20% sodium carbonate solution (Na_2_CO_3_) was added and incubated for 2 h in the dark, at room temperature. The blank was prepared in the same manner, using water to replace the chamomile extract. The absorbance was determined using a UV–visible spectrophotometer at 765 nm. The standard curve of gallic acid was obtained using the same procedure.

### 4.6. Radical Scavenger Capacity

Radical scavenger capacity was assessed by two different tests: (a) DPPH (2,2-diphenyl-1-picryl-hydrazyl-hydrate) free radical method is an antioxidant assay based on electron transfer that produces a violet solution in ethanol. A color change is noted if antioxidants are present in the analyzed sample at 517 nm; (b) Reduction of blue-green ABTS radical colored solution by hydrogen-donating antioxidants was measured by the suppression of its characteristic long wave (734nm) absorbance spectrum. Both methods were previously described by Cioanca et al. [51]. Positive controls were obtained from standard quercetin and gallic acid of high purity grade. The scavenging activity (A%) was calculated according to the formula A% = 100 × (Econtrol − Esample)/(Econtrol), where Econtrol is the absorbance of the control solution, whereas Esample is the absorbance of the solution measured in the presence of plant extracts or standards.

### 4.7. Iron Chelation Potential

The intensity given by ferrozine-Fe^2+^ complexes in the presence of iron chelating agents from plant extract is measured at 562 nm. Briefly, 2 mM iron chloride solution was added to a test tube containing a variable amount of investigated extracts (0.078125–10 mg/mL), DMSO (400 µL), and 5 mM ferrozine (80 µL). The standard was represented by caffeic acid of high purity grade (Sigma). By linearly plotting the inhibition percentage against sample concentration, we obtained EC_50_ (sample concentration providing 50% inhibition of ferrozine).

### 4.8. Lipoxygenase Inhibition Activity

An aliquot (0.05 mL) of 15-lipoxygenase in borate buffer (pH 9) was mixed with 0.05 mL of the sample solution in DMSO (various concentrations); 10 min later, 2 mL of 0.16 mM linoleic acid borate buffer were added, and the absorbances were registered at 234 nm for 90 s [26]. Inhibition of 15-lipoxygenase was established with the formula: % inhibition = (AEFI − AECI) × 100/AEFI; AEFI is the difference of the enzyme absorbance without inhibitor at 90 and 30 s, while AECI represents the same difference of the enzyme–inhibitor mixture.

### 4.9. Cell Culture and Preparation

The A375 human melanoma cell line (ECACC; Sigma Aldrich origin Japan stored UK) was cultured in minimal essential medium supplemented with 10% fetal bovine serum, 1% antibiotic/antimycotic (penicillin/streptomycin) mixture, and 1% non-essential amino acids, in humidified air containing 5% CO_2_ at 37 °C. All the medium and supplements were purchased from Lonza Group Ltd. (Basel, Switzerland). Human dendritic cells were differentiated from isolated PBMCs from buffy coats according to Nair et al. [52]. In brief, density centrifugation was performed using Ficoll (GE Healthcare, Uppsala, Sweden). Isolated PBMCs have been plated at a density of 2 × 10^8^ cells per dish, and supernatant has been discarded upon 2 h of plastic adherence. Subsequently, cells were differentiated in RPMI 1640 GlutaMax medium (Thermo Fisher Scientific, Waltham, MA, USA) supplemented with 10% FCS, 100 IU/mL penicillin, 100 µg/mL streptomycin, 10 mM HEPES (Sigma-Aldrich, Steinheim, Germany), 1 mM sodium pyruvate, and 50 µM 2-β-ME (Thermo Fisher Scientific, Massachusetts, MA, USA) supplemented with 40 ng/mL recombinant human GM-CSF (Peprotech, Rocky Hill, NJ, USA), and human IL4 (Peprotech, Rocky Hill, NJ, USA), with an additional medium exchange after 4 days. Differentiated cells were harvested by cell scraping, and transferred to 6-well plates for further experiments, or to tissue-treated 8-well chambered coverslides (Ibidi, Martinsried, Germany) for fluorescence microscopy staining. The supernatants were analyzed by ELISAs for TNF-alpha, IL-10, and IL-6 (R&D Systems, Wiesbaden, Germany), according to the manufacturer’s manual.

### 4.10. Antiproliferative Activity Assays

The growth-inhibitory activity of the extracts against A375 cells was determined by the well-known MTT assay. Experiments were carried out in the same way as previously described [53]. Briefly, cells were plated into 96-well plates at a density of 5000 cells/well, and incubated with selected concentrations of extracts (10, 30, 60 µg/mL) for 72 h. After this period, 5 mg/mL MTT (3-(4,5-dimethylthiazol-2-yl)-2,5-diphenyltetrazolium bromide) solution was added, followed by another 4 h of incubation. Dimethyl sulfoxide (DMSO) was used in order to dissolve the crystals of precipitated formazan. The absorbance was measured using a microplate reader at 545 nm. Wells with vehicle-treated cells were used as control [54].

The XTT Cell Viability Assay (Thermo Fischer Scientific) was used, according to the manufacturer’s manual, on human dendritic cells. In brief, the final XTT solution was added to cell culture wells with cells, or medium only as a control. Upon 45 min incubation time at 5% CO_2_ and 37 °C, aliquots of the cells were analyzed in flat 96-well plates (Greiner, Frickenhausen, Germany) at 460, and normalized to 650 nm. In advance, cells have been stimulated with vehicle, or tested extracts in the presence or absence of LPS for 48 h at 60 µg/mL. Cell number was obtained after XTT assay and normalized to 10^4^ cells.

### 4.11. Cell Cycle Analysis by Flow Cytometry

In order to measure the distribution of DNA between the phases of the cell cycle following incubation with selected extracts, flow cytometric analysis was performed [55]. A375 human melanoma cells were seeded into 6-well plates at a density of 300,000–400,000 cells/well. After 24 h of treatment with selected extract concentrations (30 or 60 µg/mL), the cells were washed twice with cold phosphate-buffered saline (PBS), harvested with trypsin, and centrifuged at 1500 rpm for 10 min. After a washing step, the cells were fixed in 1 mL 70% ethanol, at −20 °C, for 30 min. Samples were stained with 1.0 mL dye solution containing 0.02 mg/mL RNase A, 0.1 mg/mL PI, 0.003 mL/mL Triton-X, and 1.0 mg/ mL sodium citrate in distilled water, and the mixture was incubated in the dark for 60 min at room temperature. Cells were analyzed by a Partec CyFlow instrument (Partec GmbH, Münster, Germany). In each analysis, 20,000 events were recorded, and the percentages of the cells in the different cell cycle phases (subG1, G1, S and G2/M) were determined by using ModFit Software (Version 5 Verity Software House, Topsham, ME, USA).

### 4.12. Determination of the In Situ Caspase Activity

For the determination of the in situ caspase 3 activity, a commercially available colorimetric assay (Casp-3-C, Sigma-Aldrich, Budapest, Hungary) was performed. Cells (10^7^) were seeded in tissue culture flasks for control, and 10^12^ cells were seeded for treatment. On the second day, the cells were treated with selected concentrations of extracts (30 or 60 µg/mL). After incubation for 48 h, cells were counted, centrifuged, and washed with PBS. The cells were resuspended in kit lysis buffer at a concentration of 10^7^ cells per 100 mL, and incubated on ice for 20 min. The lysate was centrifuged, and the supernatant was used. The protein concentration of the lysate was measured with the Pierce BCA Protein Assay Kit (Pierce Biotechnology, Rockford, IL, USA), and equal amounts of protein were used for the measurement. In accordance with the manufacturer’s protocol, 5.0 µL portions of treated and untreated cell lysates were incubated with 10 µL selective caspase 3 substrate (acetyl–Asp–Glu–Val–Asp–*p*-nitroanilide) in a final volume of 100 µL in assay buffer, in 96-well plates. After an overnight incubation at 37 °C, the absorbance of *p*-nitroaniline was measured at 405 nm with a microplate reader. Comparison of the absorbances of the *p*-nitroaniline from treated and untreated samples allowed determination of the fold increase in caspase 3 activity.

### 4.13. Annexin V/PI Assay

The evaluation of early and late apoptosis, as well as necrosis, was conducted with selected concentrations of extracts (30, 60 µg/mL). Cells at 5 × 10^5^ cells/well were seeded into 6-well plate (Greiner bio-one, Gmbh, Germany) and left overnight, in order to attach to the bottom of the plate. After 24 h, the cultured medium (DMEM) was removed, and a fresh medium containing the tested extracts was added. Untreated cells were used as control; cells treated with DMSO were used as solvent control. After 72 h, cells were trypsinized. Annexin V-FITC combined with propidium iodide (PI) kit (Invitrogen, ThermoFisher, Vienna, Austria) was used in cell death flow cytometric studies (apoptosis) following the manufacturer’s protocol. Briefly, 2–5 × 10^5^ cells were washed twice in 1 × Annexin V Binding Buffer, centrifuged at 1500 rpm for 5 min, resuspended in the binding buffer and incubated with 5 μL of Annexin V-FITC for 15 min in the dark. After washing the cells with 200 μL specific binding buffer and centrifugation, the cell pellet was resuspended in 190 μL binding buffer, and 10 μL of PI solution was added immediately prior to analysis by flow cytometry.

### 4.14. Immunocytochemistry

Immunocytochemistry was performed in order to evaluate the expression of protein caspase 2 and cellular tumor antigen p5. Cells were plated at a density of 10,000 cells/cm^2^ in 4-well glass chamber slides (Nalgene Nunc International, New York, NY, USA) and expanded for 24 h in culture medium. After 24 h, the tested extracts were added. Untreated cells were used as control. After a 72 h period of incubation with tested extracts, cells were prepared for immunocytochemical staining. The culture medium was removed, and cells were washed, and fixed and permeabilized for 10 min with methanol (Sigma-Aldrich) at −20 °C. After fixation and permeabilization, cells were analyzed for the expression of the proteins of interest, anti-h/m caspase 2 (mCaspase 2 affinity purified rabbit IgG) (R&D Systems, Abingdon, UK) and p53 (monoclonal mouse anti-human) (Dako, Carpinteria, CA, USA). The staining protocol used the secondary biotinylated antibody binding and substrate addition (AEC) (Dako EnVision™ + System-HRP, Dako, Carpinteria, CA, USA) following the manufacturer’s protocol. After counterstaining with hematoxylin solution (Hematoxylin, Mayer’s Lillie’s Modification, Dako, Carpinteria, CA, USA) for 10 min, and washing with tap water, the slides were mounted in an aqueous mounting media (Crystal/Mount™, Biomeda, Foster City, CA, USA). Microscopy analysis was performed on a Zeiss Axio Observer D1 microscope.

### 4.15. Fluorescence Microscopy

Cells were grown on tissue-treated 8-well chambered coverslides (Ibidi, Martinsried, Germany), cultured, and fixed as described before [56]. DAPI (4′,6-diamidine-2′-phenylindole dihydrochloride) solution (Roche Diagnostics, Mannheim, Germany) and phalloidin Alexa Fluor 488 solution (Thermo Fisher) was applied for 1 h. Confocal laser scanning microscopy was performed with a Zeiss LSM510 Meta system equipped with an inverted Observer Z1 microscope and a Plan-Apochromat 63 ×/1.4 oil immersion objective (Carl Zeiss MicroImaging GmbH, Göttingen, Germany).

### 4.16. Statistics

The Prism software package GraphPad Prism 5.01 (GraphPad Software, San Diego, CA, USA) was used for data collection and presentation. The data ranged from three to five separate experiments, and are presented as mean ± SD. Statistical significance was assessed by one-way ANOVA with Newman–Keuls post-test for comparison of multiple groups, where *, **, ***, and **** indicate *p* < 0.05, *p* < 0.01, *p* < 0.001, and *p* < 0.0001, respectively, compared to the control group.

## 5. Conclusions

The phytochemical analysis showed that chamomile, parsley, and celery methanol extracts contain natural compounds that belong to the polyphenolic acids and flavone groups. Apigenin in the form of aglycone, as well as heteroside, could be detected in all extracts, but it is clearly visible that the seeds contain mainly aglycons and less glycosides. The tested samples showed radical scavenger capacity, iron chelation potential, as well as lipoxygenase inhibition capacity. The three extracts showed an overall weak antiproliferative activity against A375 human melanoma cell line in the range of tested concentrations. Among the screened extracts, parsley was the most active in terms of pro-apoptotic properties, inducing both caspase 3 and caspase 2, as well as phenomena of early apoptotic, late apoptotic, and necrotic cells. Parsley and chamomile extracts affected the fibroblast-like morphology of A375 human melanoma cells. Regarding the activity on human dendritic cells, chamomile and celery extracts abrogated the expansion of LPS-activated dendritic cells. On the other hand, the metabolic activity of active human DCs was attenuated by stimulation with celery extract, while chamomile and parsley extracts had no effect on the metabolic activity. Extract incubation with naïve dendritic cells did not trigger cytokine secretion (TNF-alpha, IL-6, IL-10), indicating that the extracts themselves have no immune reactivity in the given settings, only the levels of anti-inflammatory cytokine IL-10 being significantly reduced upon celery extract stimulation.

## Figures and Tables

**Table 1:** The major polyphenolic compounds of the investigated plant samples by RP-UHPL (µg/100 µg extract).

	Chlorogenic Acid	Caffeic Acid	Catechin	Apigenin Glucoside	Rutin	Cinaroside	Luteolin	Apigenin	Kaempferol
MC extract	8.180	1.296	5.179	34.103	6.013	2.659	5.113	1.388	-
P extract	4.572	2.213	-	22.371	-	0.776	4.078	14.562	2.662
C extract	6.609	1.980	-	18.015	-	6.146	1.539	16.967	8.480

“-” not detected.

**Table 2:** Spectrophotometric quantification of flavonoids and total phenols in investigated extracts.

Sample	Total Flavones (µg /mL Extract)	Total Phenols (µg GAE/mL Extract)
MC extract	342.4	458.1
P extract	172.2	292.7
C extract	229.3	345.2

**Table 3:** Radical scavenger capacity of the investigated extracts.

Extract/Standard	EC_50_ (µg/mL)	
	DPPH Assay	ABTS Assay
MC extract	47.8 ± 1.4	21.4 ± 0.2
C extract	157.9 ± 2.1	163.7 ± 1.3
P extract	165.4 ± 0.2	164.1 ± 1.5
Caffeic Acid	3.6 ± 0.0	1.6 ± 0.0

**Table 4:** Iron chelation potential of the selected extracts.

Sample	0.078125 mg/mL	0.15625 mg/mL	0.3125 mg/mL	0.625 mg/mL	1.25 mg/mL	2.5 mg/mL	5 mg/mL	10 mg/mL	EC_50_ (mg/mL Solution Final Tube)
MC extract	3.12 ± 0.05	4.77 ± 0.05	10.25 ± 0.12	13.28 ± 0.10	25.41 ± 0.07	50.59 ± 0.12	69.89 ± 0.13	73.91 ± 0.02	491.94 ± 1.61
C extract	2.25 ± 0.03	3.00 ± 0.04	7.73 ± 0.04	19.91 ± 0.03	51.44 ± 0.01	88.15 ± 0.03	91.65 ± 0.04	92.96 ± 0.01	242.21 ± 0.06
P extract	6.84 ± 0.09	10.75 ± 0.04	12.39 ± 0.07	16.61 ± 0.13	24.21 ± 0.06	74.10 ± 0.02	93.55 ± 0.01	96.91 ± 0.03	357.72 ± 0.19
Caffeic Acid	97.03 ± 0.09	97.09 ± 0.14	98.36 ± 0.21	98.61 ± 0.32	99.27 ± 0.22	100 ± 0.00	100 ± 0.00	-	2.47 ± 0.25

“-” not detected.

**Table 5:** Lipoxygenase inhibition activity of selected extracts.

Sample	0.078125 mg/mL	0.15625 mg/mL	0.3125 mg/mL	0.625 mg/mL	1.25 mg/mL	2.5 mg/mL	5 mg/mL	10 mg/mL	EC_50_ (mg/mL Solution Final Tube)
MC extract	4.71 ± 0.03	7.13 ± 0.54	12.30 ± 0.30	23.85 ± 0.37	35.40 ± 0.48	43.03 ± 0.31	59.95 ± 0.28	73.38 ± 0.20	166.32 ± 2.03
C extract	7.88 ± 0.28	12.84 ± 0.17	21.30 ± 0.89	30.06 ± 0.78	44.66 ± 0.97	56.22 ± 0.89	77.01 ± 0.66	100 ± 0.00	86.15 ± 4.82
P extract	7.44 ± 0.48	10.44 ± 0.34	16.73 ± 0.64	28.08 ± 0.30	46.08 ± 0.91	71.84 ± 0.93	80.87 ± 0.72	100 ± 0.00	69.46 ± 1.70
Caffeic Acid	95.13 ± 0.79	96.15 ± 0.48	97.53 ± 0.21	98.22 ± 0.32	99.27 ± 0.17	99.43 ± 0.93	100 ± 0.00	100 ± 0.00	1.24 ± 0.05

**Table 6:** Apoptotic events for A375 human melanoma cell line after incubation with selected extracts.

	Viable Cells	Early Apoptotic Cells	Late Apoptotic Cells	Necrotic Cells
Control	95.40 ± 1.89	3.10 ± 1.62	0.67 ± 0.21	0.83 ± 0.05
MC extract (60 μg/mL)	95.76 ± 0.16	1.50 ± 0.08	0.64 ± 0.08	2.1 ± 0.16
C extract (60 μg/mL)	95.19 ± 0.95	2.38 ± 0.60	0.82 ± 0.37	1.70 ± 1.17
P extract (60 μg/mL)	89.00 ± 0.09	7.30 ± 0.65	2.36 ± 0.24	1.34 ± 1.25

## Abbreviations

FDA	food and drug administration
UHPC	ultra-high-performance liquid chromatography
GAE	gallic acid equivalents
DPPH	2,2-diphenyl-1-picrylhydrazy
TPC	total phenolic content
ABTS	2,2′-azino-bis(3-ethylbenzothiazoline-6-sulphonic acid)
DMSO	dimethyl sulfoxide
PBMC	peripheral blood mononuclear cell
ELISA	enzyme-linked immunosorbent assay
MTT	3-(4,5-dimethylthiazol-2-yl)-2,5-diphenyltetrazolium bromide
XTT	2,3-bis-(2-methoxy-4-nitro-5-sulfophenyl)-2H-tetrazolium-5-carboxanilide
DAPI	4′,6-diamidino-2-phenylindole
PI	propidium iodide
MC	chamomile extract
P	parsley extract
C	celery extract
LPS	lipopolysaccharide
TNF	tumor necrosis factor
IL	interleukin
EC_50_	efficient concentration to chelate 50% of the free radicals present in the assay environment
DCs	dendritic cells
LOX	lipoxygenase

## References

[1] Ji H.F., Li X.J., Zhang H.Y. (2009). Natural products and drug discovery. Can thousands of years of ancient medical knowledge lead us to new and powerful drug combinations in the fight against cancer and dementia?. EMBO Rep..

[2] Soica C., Trandafirescu C., Danciu C., Muntean D., Dehelean C., Simu G. (2014). New Improved Drug Delivery Technologies for Pentacyclic Riterpenes: A Review. Protein Peptide Lett..

[3] Xie T., Song S., Li S., Ouyang L., Xia L., Huang J. (2015). Review of natural product databases. Cell Prolif..

[4] Robert C., Long G.V., Brady B., Dutriaux C., Maio M., Mortier L. (2015). Nivolumab in Previously Untreated Melanoma without BRAF Mutation. N. Engl. J. Med..

[5] Danciu C., Falamas A., Dehelean C., Soica C., Radeke H., Barbu-Tudoran L., Bojin F., Cîntă Pînzaru S., Munteanu M.F. (2013). A characterization of four B16 murine melanoma cell sublines: Molecular fingerprint and proliferation behavior. Cancer Cell Int..

[6] Da Rocha A.B., Lopes R.M., Schwartsmann G. (2001). Natural products in anticancer therapy. Curr. Opin. Pharmacol..

[7] Danciu C., Oprean C., Coricovac D.E., Andreea C., Cimpean A., Radeke H., Soica C., Dehelean C. (2015). Behaviour of four different B16 murine melanoma cell sublines: C57BL/6J skin. Int. J. Exp. Pathol..

[8] Chahar M.K., Sharma N., Dobhal M.P., Joshi Y.C. (2011). Flavonoids: A versatile source of anticancer drugs. Pharmacogn. Rev..

[9] Balázs B., Sipos P., Danciu C., Avram S., Soica C., Dehelean C., Varju G., Erős G., Budai-Szűcs M., Berkó S. (2016). An ATR-FTIR and Raman spectroscopic investigation of the electroporation-mediated transdermal delivery of a nanocarrier system containing an antitumour drug. Biomed. Opt. Express.

[10] Caltagirone S., Rossi C., Poggi A., Ranelletti F.O., Natali P.G., Brunetti M. (2000). Flavonoids apigenin and quercetin inhibit melanoma growth and metastatic potential. Int. J. Cancer.

[11] Hasnat M.A., Pervin M., Lim J.H., Lim B.O. (2015). Apigenin Attenuates Melanoma Cell Migration by Inducing Anoikis through Integrin and Focal Adhesion Kinase Inhibition. Molecules.

[12] Zhao G., Han X., Cheng W., Ni J., Zhang Y., Lin J. (2017). Apigenin inhibits proliferation and invasion, and induces apoptosis and cell cycle arrest in human melanoma cells. Oncol. Rep..

[13] Jeffrey C., Kubitzki K. (2007). Compositae: Introduction with key to tribes. The Families and Genera of Vascular Plants.

[14] Srivastava J.K., Gupta S. (2009). Extraction, Characterization, Stability and Biological Activity of Flavonoids Isolated from Chamomile Flowers. Mol. Cell. Pharmacol..

[15] Park E.H., Bae W.Y., Eom S.J., Kim K.T., Paik H.D. (2017). Improved antioxidative and cytotoxic activities of chamomile (*Matricaria chamomilla*) florets fermented by Lactobacillus plantarum KCCM 11613P. J. Zhejiang Univ. Sci. B.

[16] Srivastava J.K., Gupta S. (2007). Antiproliferative and apoptotic effects of chamomile extract in various human cancer cells. J. Agric. Food Chem..

[17] Downie S.R., Katz-Downie D.S., Watson M.F. (2000). A phylogeny of the flowering plant family Apiaceae based on chloroplast DNA rpl16 and rpoC1 intron sequences: Towards a suprageneric classification of subfamily Apioideae. Am J. Bot..

[18] Farzaei M.H., Abbasabadi Z., Ardekani M.R.S., Rahimi R., Farzaei F. (2013). Parsley: A review of ethnopharmacology, phytochemistry and biological activities. J. Tradit. Chin. Med..

[19] Tang E.L., Rajarajeswaran J., Fung S., Kanthimathi M. (2015). Petroselinum crispum has antioxidant properties, protects against DNA damage and inhibits proliferation and migration of cancer cells. J. Sci. Food Agric..

[20] Kooti W., Daraei N. (2017). A Review of the Antioxidant Activity of Celery (*Apium graveolens* L.). Evid. Based Complement. Altern Med..

[21] Powanda M.C., Whitehouse M.W., Rainsford K.D. (2015). Celery Seed and Related Extracts with Antiarthritic, Antiulcer, and Antimicrobial Activities. Prog. Drug Res..

[22] Mansi K., Abushoffa A.M., Disi A., Aburjai T. (2009). Hypolipidemic Effects of Seed Extract of Celery (*Apium graveolens*) in Rats. Pharmacogn. Mag..

[23] Powanda M.C., Rainsford K.D. (2011). A toxicological investigation of a celery seed extract having anti-inflammatory activity. Inflammopharmacology.

[24] Quassinti L., Maggi F., Barboni L., Ricciutelli M., Cortese M., Papa F. (2014). Wild celery (*Smyrnium olusatrum* L.) oil and isofuranodiene induce apoptosis in human colon carcinoma cells. Fitoterapia.

[25] Malterud K.E., Farbrot T.L., Huse A.E., Sund R.B. (1993). Antioxidant and radical-scavenging effects of anthraquinones and anthrones. Pharmacology.

[26] Srivastava J.K., Shankar E., Gupta S. (2010). Chamomile: A herbal medicine of the past with bright future. Mol. Med. Rep..

[27] Svehlíková V., Wang S., Jakubíková J., Williamson G., Mithen R., Bao Y. (2004). Interactions between sulforaphane and apigenin in the induction of UGT1A1 and GSTA1 in CaCo-2 cells. Carcinogenesis.

[28] Mojzer E.B., Hrnčič M.K., Škerget M., Knez Z., Bren U. (2016). Polyphenols: Extraction methods, antioxidative action, bioavailability and anticarcinogenic effects. Molecules.

[29] Haghi G., Hatami A., Safaei A., Mehran M. (2014). Analysis of phenolic compounds in Matricaria chamomilla and its extracts by UPLC-UV. Res. Pharm. Sci..

[30] Fonseca F.N., Tavares M.F. (2004). Validation of a capillary electrophoresis method for the quantitative determination of free and total apigenin in extracts of *Chamomilla recutita*. Phytochem. Anal..

[31] Bhaskaran N., Shukla S., Srivastava J.K., Gupta S. (2010). Chamomile, an anti-inflammatory agent inhibits inducible nitric oxide synthase expression by blocking RelA/p65 activity. Int. J. Mol. Med..

[32] Cvetanović A., Švarc-Gajić J., Zeković Z., Savić S., Vulić J., Mašković P., Ćetković G. (2015). Comparative analysis of antioxidant, antimicrobiological and cytotoxic activities of native and fermented chamomile ligulate flower extracts. Planta.

[33] Al-Ismail K.M., Aburjai T. (2004). Antioxidant activity of water and alcohol extracts of chamomile flowers, anise seeds and dill seeds. J. Sci. Food Agric..

[34] Jabri M.A., Aissani N., Tounsi H., Sakly M., Marzouki L., Sebai H. (2017). Protective effect of chamomile (*Matricaria recutita* L.) decoction extract against alcohol-induced injury in rat gastric mucosa. Pathophysiology.

[35] Yıldız L., Başkan K.S., Tütem E., Apak R. (2008). Combined HPLC-CUPRAC (cupric ion reducing antioxidant capacity) assay of parsley, celery leaves, and nettle. Talanta.

[36] Wong P.Y.Y., Kitts D.D. (2006). Studies on the dual antioxidant and antibacterial properties of parsley (*Petroselinum crispum*) and cilantro (*Coriandrum sativum*) extracts. Food Chem..

[37] Yao Y., Ren G. (2011). Effect of thermal treatment on phenolic composition and antioxidant activities of two celery cultivars. LWT-Food Sci. Technol..

[38] Vranješ M., Popovic’ B.M., Štajner D., Ivetic V., Mandic A., Vranješ D. (2016). Effects of bearberry, parsley and corn silk extracts on diuresis, electrolytes composition, antioxidant capacity and histopathological features in mice kidneys. J. Funct. Foods.

[39] Csepregi K., Neugart S., Schreiner M., Hideg É. (2016). Comparative evaluation of total antioxidant capacities of plant polyphenols. Molecules.

[40] Tanasawet S., Boonruamkaew P., Sukketsiri W., Chonpathompikunlert P. (2017). Anxiolytic and free radical scavenging potential of Chinese celery (*Apium graveolens*) extract in mice. Asian Pac. J. Trop. Biomed..

[41] Sak K., Nguyen T.H., Ho V.D., Do T.T., Raal A. (2017). Cytotoxic effect of chamomile (*Matricaria recutita*) and marigold (*Calendula officinalis*) extracts on human melanoma SK-MEL-2 and epidermoid carcinoma KB cells. Cogent Med..

[42] Matić I.Z., Juranić Z., Šavikin K., Zdunić G., Nađvinski N., Gođevac D. (2012). Chamomile and Marigold Tea: Chemical Characterization and Evaluation of Anticancer Activity. Phytother. Res..

[43] Ahmad R., Ahmad N., Naqvi A.A., Shehzad A., Al-Ghamdi M.S. (2017). Role of traditional Islamic and Arabic plants in cancer therapy. J. Tradit. Complement. Med..

[44] Subhadradevi V., Khairunissa K., Asokkumar K., Thirumalaisamy S., Jagannath P. (2011). Induction of Apoptosis and Cytotoxic Activities of Apium graveolens Linn. Using in vitro Models. Middle-East J. Sci. Res..

[45] Zidorn C., Jöhrer K., Ganzera M., Schubert B., Sigmund E.M., Mader J. (2005). Polyacetylenes from the Apiaceae Vegetables Carrot, Celery, Fennel, Parsley, and Parsnip and Their Cytotoxic Activities. J. Agric. Food Chem..

[46] Sultana S., Ahmed S., Jahangir T., Sharma S. (2005). Inhibitory effect of celery seeds extract on chemically induced hepatocarcinogenesis: Modulation of cell proliferation, metabolism and altered hepatic foci development. Cancer Lett..

[47] Dorman H.J.D., Lantto T.A., Raasmaja A., Hiltunen R. (2011). Antioxidant, pro-oxidant and cytotoxic properties of parsley. Food Funct..

[48] Schröder L., Koch J., Mahner S., Kost B.P., Hofmann S., Jeschke U., Haumann J., Schmedt J., Richter D.U. (2017). The Effects of Petroselinum Crispum on Estrogen Receptor-positive Benign and Malignant Mammary Cells (MCF12A/MCF7). Anticancer Res..

[49] Iancu C., Cioancă O., Mircea C., Mocanu M., Hăncianu M. (2016). *Pelargonium* sp.: Characterization of the Polyphenols and their Biological Potential. Farmacia.

[50] Mircea C., Cioancă O., Iancu C., Tătărînga G., Hăncianu M. (2015). In Vitro Antioxidant Activity of some Extracts Obtained from *Agaricus bisporus* brown. *Pleurotus ostreatus* and *Fomes fomentarius*. Farmacia.

[51] Cioancă O., Pagonakis A., Trifan A., Hrițcu L., Ioniţă R., Burlec A.F., Postu P., Cornelia M., Hăncianu M. (2017). Pharmacognostic and pharmacologic screening of crocus sativus of greek origin. Farmacia.

[52] Nair S., Archer G.E., Tedder T.F. (2012). Isolation and generation of human dendritic cells. Curr. Protoc. Immunol..

[53] Szabó J., Jerkovics N., Schneider G., Wölfling J., Bózsity N., Minorics R., Zupkó I., Mernyák E. (2016). Synthesis and in vitro antiproliferative evaluation ofC-13 epimers of triazolyl-d-secoestrone alcohols: The first potent 13a-D-secoestrone derivative. Molecules.

[54] Mosmann T. (1983). Rapid colorimetric assay for cellular growth and survival: Application to proliferation and cytotoxicity assays. J. Immunol. Methods.

[55] Vermes I., Haanen C., Reutelingsperger C. (2000). Flow cytometry of apoptotic cell death. J. Immunol. Methods.

[56] Schwiebs A., Thomas D., Kleuser B., Pfeilschifter J.M., Radeke H.H. (2017). Nuclear translocation of SGPP-1 and decrease of SGPL-1 activity contribute to sphingolipid rheostat regulation of inflammatory dendritic cells. Mediat. Inflamm..

